# *Trypanosoma cruzi* infection in *Triatoma infestans* and high levels of human–vector contact across a rural-to-urban gradient in the Argentine Chaco

**DOI:** 10.1186/s13071-020-04534-z

**Published:** 2021-01-09

**Authors:** Alejandra Alvedro, María Sol Gaspe, Hannah Milbourn, Natalia Paula Macchiaverna, Mariano Alberto Laiño, Gustavo Fabián Enriquez, Ricardo Esteban Gürtler, Marta Victoria Cardinal

**Affiliations:** 1 Laboratorio de Eco-Epidemiología, Facultad de Ciencias Exactas y Naturales, Universidad de Buenos Aires, Buenos Aires, Argentina; 2 Instituto de Ecología, Genética y Evolución de Buenos Aires (IEGEBA), Consejo Nacional de Investigaciones Científicas y Técnicas–Universidad de Buenos Aires, Buenos Aires, Argentina; 3 London School of Hygiene and Tropical Medicine, London, UK

**Keywords:** *Trypanosoma cruzi*, *Triatoma infestans*, Urbanization, Blood-feeding sources

## Abstract

**Background:**

Peri-urban and urban settings have recently gained more prominence in studies on vector-borne transmission of *Trypanosoma cruzi* due to sustained rural-to-urban migrations and reports of urban infestations with triatomines. Prompted by the finding of *Triatoma infestans* across the rural-to-urban gradient in Avia Terai, an endemic municipality of the Argentine Chaco, we assessed selected components of domestic transmission risk in order to determine its variation across the gradient.

**Methods:**

A baseline vector survey was conducted between October 2015 and March 2016, following which we used multistage random sampling to select a representative sample of *T. infestans* at the municipal level. We assessed *T. cruzi* infection and blood-feeding sources of 561 insects collected from 109 houses using kinetoplast DNA-PCR assays and direct enzyme-linked immunosorbent assays, respectively. We stratified triatomines according to their collection site (domestic or peridomestic), and we further categorized peridomestic sites in ecotopes of low- or high-risk for *T. cruzi* infection.

**Results:**

The overall adjusted prevalence of *T. cruzi*-infected* T. infestans* was 1.8% (95% confidence interval [CI] 1.3–2.3) and did not differ between peri-urban (1.7%) and rural (2.2%) environments. No infection was detected in bugs captured in the urban setting; rather, infected triatomines were mainly collected in rural and peri-urban domiciles, occurring in 8% of* T. infestans*-infested houses. The main blood-feeding sources of domestic and peridomestic triatomines across the gradient were humans and chickens, respectively. The proportion of triatomines that had fed on humans did not differ between peri-urban (62.5%) and rural (65.7%) domiciles, peaking in the few domestic triatomines collected in urban houses and decreasing significantly with an increasing proportion of chicken- and dog- or cat-fed bugs. The relative odds ratio (OR) of having a *T. cruzi* infection was nearly threefold higher in bugs having a blood meal on humans (OR 3.15), dogs (OR 2.80) or cats (OR: 4.02) in a Firth-penalized multiple logistic model.

**Conclusions:**

*Trypanosoma cruzi* transmission was likely occurring both in peri-urban and rural houses of Avia Terai. Widespread infestation in a third of urban blocks combined with high levels of human–triatomine contact in the few infested domiciles implies a threat to urban inhabitants. Vector control strategies and surveillance originally conceived for rural areas should be tailored to peri-urban and urban settings in order to achieve sustainable interruption of domestic transmission in the Chaco region.
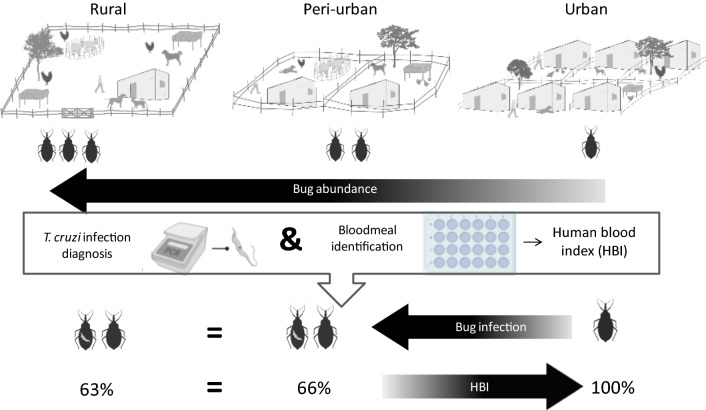

## Introduction

Chagas disease, a neglected tropical disease endemic to the Americas, affects about 6–7 million people [1]. Its etiological agent, the protozoan *Trypanosoma cruzi*, is associated with cardiac and digestive syndromes in a third of infected patients and causes nearly 10,000 premature deaths each year [2]. Vector-borne transmission mediated by triatomine bugs has been traditionally the main route of transmission in endemic areas and still occurs in 21 countries [1].

Originally linked to impoverished rural environments where mud-and-thatch houses predominated and harbored large bug colonies, the landscape of Chagas disease has changed over the years. Migration has driven originated the globalization and urbanization of Chagas disease, with big cities worldwide now witnessing both the occurrence of congenital transmission [3] and of infected people [4, 5]. This phenomenon is not unique to Chagas disease; other diseases, such as visceral leishmaniasis and schistosomiasis, are experiencing the same shifting epidemiology [6–8].

Urban infestations and vector-borne transmission of *T. cruzi* have increasingly been reported in endemic regions throughout Central and South America [9–16]. In Cochabamba (Bolivia) and Arequipa (Peru), peri-urban infestations with *Triatoma infestans*, the main vector in the region, have been linked to the immigration of farmers from rural areas [13, 17]. In these peri-urban areas, *T. cruzi* infection in *T. infestans* ranged from 14 to 19% and was associated with the presence of guinea pigs [10, 17].

Domestic *T. cruzi* transmission to humans depends on the contact rates between infected triatomines and susceptible hosts. Ecological, environmental, socio-economic and cultural factors favor or hinder these encounters [18]. Housing type, building materials, husbandry practices and the number and density of domestic hosts may boost triatomine population size and hence facilitate transmission [19, 20]. The presence and increased number of *T. cruzi*-infected dogs and cats (the main domestic reservoir hosts) increase the infection risk of householders [21]. On the contrary, appropriate domestic animal management, regular cleaning activities, domestic insecticide use, window or door screening and plastering are protective factors [22–24].

As part of an intervention program aimed at suppressing house infestation with *T. infestans* in a sustainable manner and reducing disease burden in Avia Terai, Argentine Chaco, we conducted a survey that found a decreasing prevalence of house infestation (i.e. domestic or/and peridomestic infestation) with *T. infestans*, ranging from 42.4% in rural houses to 22.5 and 4.5% in peri-urban and urban areas, respectively [25]. Domestic infestation, defined as the occurrence of *T. infestans* in human sleeping quarters or domiciles, also decreased across the rural-to-urban gradient, from 9.9 to 1.1%, respectively, as did the household number of domestic animals and peridomestic structures. *Triatoma infestans* seemed to be long established in Avia Terai, unlike in Arequipa and Cochabamba [17, 25, 26].

In the study reported here, we evaluated three selected components of *T. cruzi* transmission risk across a rural-to-urban gradient: (i) parasite infection prevalence in domestic and peridomestic *T. infestans*; (ii) triatomine bloodmeal sources; and (iii) degree of human-vector contact via the human blood index (HBI), defined as the proportion of reactive triatomines that fed on humans [27, 28]. Based on a decreasing prevalence of house and domestic infestation with *T. infestans* across the rural-to-urban gradient, we hypothesized that the prevalence of *T. cruzi* infection in *T. infestans* and the HBI in domestic bug populations would decrease across the rural-to-urban gradient. Secondly, we hypothesized that the presence of dogs, cats and chickens would decrease the HBI throughout the three environments.

## Methods

### Study area

Fieldwork was conducted in the municipality of Avia Terai (26°42′ S, 60°44′ W), Chaco Province, Argentina, which has been described elsewhere [25]. The last house spraying campaign with pyrethroid insecticides conducted by Chagas vector control personnel was carried out in 2011–2013 (2–4 years prior to our survey) and did not achieve full coverage. For the purpose of this study, we divided the municipality into three environments based on house arrangement patterns: urban (blocks), peri-urban (blocks and dispersed houses) and rural (dispersed houses) [25]. The peri-urban environment was highly heterogeneous and was further subclassified into established and recent neighborhoods [25].

### Study design

A cross-sectional survey was conducted in all environments to evaluate house and domestic infestation with triatomines at baseline between October 2015 and March 2016, as described elsewhere [25]. In total, 2296 inhabited houses were surveyed. Domestic and peridomestic ecotopes were searched for triatomines if dwellers had reported having seen triatomines or had peridomestic structures housing animals, and in one of every three houses that did not fulfil both conditions. Timed manual searches for bugs were performed for 15 min in each domestic and peridomestic structure by technicians of the Chagas control program with the aid of a flushing-out agent (0.2% tetramethrin; Espacial, Buenos Aires, Argentina). All rural houses and infested houses and their adjacent ones in peri-urban and urban environments were immediately sprayed with insecticide after the search for triatomines. A standard dose of beta-cypermethrin (50 mg/m^2^; Sipertrin, Chemotecnica, Argentina) in domiciles and a double dose in peridomiciles were used to afford prolonged residual efficacy. Recently built peri-urban neighborhoods were excluded from the present analysis since no triatomines were found there [25].

Additional bug collections were attempted by technicians during and after insecticide applications or after the timed manual searches, and separately by householders; these insects were kept separately and labeled accordingly. These additional bug collections were performed to increase sample sizes for other purposes or to enhance triatomine detection; they were only used for abundance estimation in five houses (see text below). All triatomines were identified to species, sex and stage, and immediately frozen at − 20 °C. Two other triatomine species captured in the study area, *Triatoma sordida* and *Panstrongylus geniculatus*, were excluded from the current analysis [25].

### Bug sampling design

The bugs collected were classified according to where they had been collected (domestic or peridomestic ecotopes). Peridomestic ecotopes were further subdivided into two categories according to the probability of bug infection with *T. cruzi*: (i) those with a high risk of bug infection due to the presence of dogs, cats and rodents, including kitchens, granaries, mud ovens, storerooms and kennels; and (ii) those with a low risk of bug infection, mainly associated with poultry, which are refractory to *T. cruzi* (i.e. chicken nests, trees where chickens roosted, chicken coops) and goat and pig corrals.

A multistage sampling plan was used to select bugs for dissection. Only third-instar nymphs to adult bugs were examined. In the urban environment, we examined all triatomines collected in domiciles and high-risk peridomestic ecotopes, whereas in the peri-urban and rural environments we randomly selected up to ten and five triatomines per site, respectively. Additionally, in the three environments we randomly selected 25% of infested houses with low-risk peridomestic ecotopes; up to 5 bugs per site were examined for infection in each of these houses. If more than one low-risk ecotope was infested at a given house, we randomly examined one ecotope. We separated the rectal ampoule, blood meal contents and the rest of each individual triatomine into labeled microtubes, as described previously [29].

### Diagnosis of *T. cruzi* infection

Rectal ampoules were preserved in 50 μl of sterile water for parasite DNA extraction, boiled for 10 min and the DNA extracted using DNAzol® (Invitrogen, Carlsbad, CA, USA), following a previously described protocol [29]. A hot-start PCR targeting a 330-bp amplicon of the kinetoplast minicircle was employed [30]. PCR products were run in a 3% agarose gel and visualized under UV employing Gel Red® (Biotium, Hayward, CA USA).

### Blood meal identification

Blood meal contents were stored in microtubes containing phosphate buffered saline with crystal violet. Identification of the blood meal was carried out using a direct enzyme-linked immunosorbent assay (ELISA) as described by Gürtler et al. [31]. The antisera employed in the ELISA assays included the most frequent (peri)-domestic blood meal sources of *T. infestans*: human, dog, cat, chicken, mice, goat and pig. We considered a bug as reactive when it was positive against any of the antisera tested in the ELISA assay and as a mixed blood meal when it was reactive to at least two different host species. The infective blood meal index was estimated as the percentage of *T. cruzi*-infected bugs with an identified blood meal on host X relative to the number of bugs examined for *T. cruzi* infection that had an identified blood meal on host X [32].

### Data analysis

Agresti-Coull binomial 95% confidence intervals (95% CI) were calculated for the percentage of houses harboring infected triatomines, bug infection prevalence and HBI. Wilson 95% CI were calculated when the number of examined bugs was < 40 or the proportions were near 0 or 1 [33]. Proportions were compared using Fisher’s exact or Pearson’s *χ*^2^ tests. Based on the observed prevalence of infection by bug collection ecotope (domicile, low-risk and high-risk peridomestic ecotope), we estimated the number of infected bugs that would have been detected if all collected third-instar nymphs and later stages had been examined. With these figures we estimated the adjusted prevalence of infection for each environment (i.e. urban, peri-urban and rural) and for the entire study area.

The relative abundance was calculated as the number of live triatomines collected during the timed manual searches per unit of search effort (i.e. 15 person-min per site). As an exception, in three rural and two peri-urban houses with examined bugs that had not been collected during the timed manual searches, we estimated bug abundance considering these additional collections.

We examined the relationship between human blood-feeding or bug infection with *T. cruzi* (binary response variables) and *a priori* selected predictors (see text below) by means of a random-intercept and a Firth-penalized logistic regression, respectively. Odds ratios (OR) and their 95% CI were calculated using the ‘sjPlot’ package [34]. Numerical variables were categorized according to their quartiles or medians. All predictors were established* a priori* based on existing empirical evidence [28, 31, 35]. Host numbers were obtained from house-to-house surveys [25].

For the HBI model, we performed the regression analyses clustered by house using the ‘lmer’ function implemented in the ‘lme4’ package for R (R Foundation for Statistical Computing, Vienna, Austria). We included as predictors: the relative abundance of domestic bugs, bug stage (with four levels: third- or fourth-instar nymphs, fifth instars, adult males and adult females), the number of human residents, the number of dogs and/or cats in the household, dog or cat blood index and the chicken blood index (expressed as proportions), and only considered reactive domestic bugs (total: 157 bugs from 35 houses).

In order to identify risk factors associated with *T. cruzi* infection in triatomines we employed univariate and multiple Firth-penalized logistic regressions implemented in the ‘logistf’ package in R (version 2.15.1) [36, 37] given that few infected bugs were detected and complete separation of our dataset was observed for the urban environment. We included as predictors: relative bug abundance, type of environment (urban, peri-urban or rural), bug stage (with four levels: third- or fourth-instar nymphs, fifth instars, adult males and adult females), the number of human residents, the number of dogs and/or cats in the household and whether or not the bug had an identified blood meal on humans, dogs, cats or chickens (binary variables). This model only included examined bugs from domiciles and high-risk peridomestic ecotopes; thus, it comprised 408 bugs from 76 houses. Low-risk peridomestic ecotopes were excluded from this model because no infected bug was found in these ectopes. We evaluated and found no significant effects of the interaction between type of environment and each explanatory variable on bug infection. No multicollinearity was detected (variance inflation factor < 3.9 for every variable). The global fit of the random-effects logistic model to the data was analyzed using the Hosmer–Lemeshow test with the ‘Resource Selection’ package [38].

The spatial distribution of bug infection was assessed through global and local point pattern analyses at the house level (rural environment) or using a combination of block and house levels for peri-urban neighborhoods. Global spatial analyses of house (rural) and house or block (peri-urban) infection were performed using the K-function implemented in Programita [39]. Random labeling was used to test the null hypothesis of random events among the fixed spatial distribution of infested houses (or blocks). We set cell sizes of 300 m for rural houses and 50 m for urban or peri-urban houses or blocks, and maximum distances of search of 15 and 0.6 km, respectively. We computed the 95% confidence envelopes using 999 simulations, as reported in Gaspe et al. [25]. Local spatial aggregation of bug infection was tested using the Getis statistic (G*) [40] implemented in PPA [41], with the same parameters as for the global analysis.

## Results

### Infection prevalence

In total, 561 *T. infestans* were selected and examined for infection from among the 4408 bugs collected (Table 1). The dissected bugs were collected from 66.7, 100 and 96.2% of the infested urban, peri-urban and rural domiciles, respectively and from 87.5, 85.7 and 89.7% of the urban, peri-urban and rural houses with infested high-risk peridomestic ecotopes (Table 1). Houses not examined for bug infection only harbored first or second-instar nymphs or bugs that were preserved for other studies (including 1 urban, 6 peri-urban and 5 rural houses).

**Table 1:** Distribution of *Trypanosoma cruzi* infection in *Triatoma infestans* by environment and type of ecotope, Avia Terai, Chaco, 2015–2016

Environment and ecotope (no. of houses inspected)^a^	Bug level			House level		
	% of bugs examined (no. collected)	% of bugs infected (no. examined)	95% CI	% examined for infection (no. infested)	% harboring infected bugs (no. examined)	95% CI
Urban (416)						
Domiciles	85.7 (14)	0.0 (12)	0.0–24.3	66.7 (3)	0.0 (2)	0.0–65.8
Peridomestic HR	100.0 (67)	0.0 (67)	0.0–5.4	87.5 (8)	0.0 (7)	0.0–32.2
Peridomestic LR	7.2 (485)	0.0 (35)	0.0–9.9	25.6 (39)	0.0 (10)	0.0–27.8
Total	20.1 (566)	0.0 (114)	0.0–3.2	38.0 (50)	0.0 (19)	0.0–16.8
Peri-urban (210)						
Domiciles	98.1 (53)	17.3 (52)	9.2–30.0	100.0 (14)	21.4 (14)	6.8–48.3
Peridomestic HR	24.3 (226)	5.5 (55)	1.9–14.9	85.7 (14)	18.2 (12)	4.0–48.9
Peridomestic LR	4.3 (974)	0.0 (42)	0.0–8.4	24.3 (37)	0.0 (9)	0.0–29.9
Total	11.9 (1253)	8.1 (149)	4.7–13.6	51.7 (60)	16.1 (31)	7.1–32.6
Rural (276)						
Domiciles	50.9 (228)	9.5 (116)	5.2–16.3	96.2 (26)	24.0 (25)	11.2–43.2
Peridomestic HR	29.6 (358)	8.5 (106)	4.4–15.6	89.7 (29)	15.4 (26)	5.8–35.2
Peridomestic LR	3.8 (2003)	0.0 (76)	0.0–4.8	26.6 (64)	0.0 (17)	0.0–18.4
Total	11.5 (2589)	6.7 (298)	4.4–10.1	57.3 (103)	13.6 (59)	7.0–25.5
Total (902)	12.7 (4408)	5.7 (561)	4.1–8.0	51.2 (213)	11.9 (109)	7.7–20.5

CI, Confidence interval

^a^Peridomestic HR, Peridomestic ecotopes with high risk of bug infection; peridomestic LR, peridomestic ecotopes with low risk of bug infection

The overall prevalence of infection was 5.7%. No infected bug was detected in the urban environment whereas no significant difference in the prevalence of infection was found between the peri-urban (8.1%) and rural (6.7%) environments (Fisher’s exact test:* df* = 1, *P* = 0.7) (Table 1). Infection prevalence peaked in domiciles, followed by high-risk peridomestic ecotopes and was non existent in low-risk peridomestic ecotopes in both peri-urban and rural environments. The adjusted prevalence of bug infection (estimated as if all collected bugs had been examined) was 1.8% (95% CI 1.3–2.3), and increased from 0% in the urban environment to 1.7% (95% CI 1.1–2.6) and 2.2% (95% CI 1.6–2.8) in the peri-urban and rural environments, respectively.

**Fig. 1:**
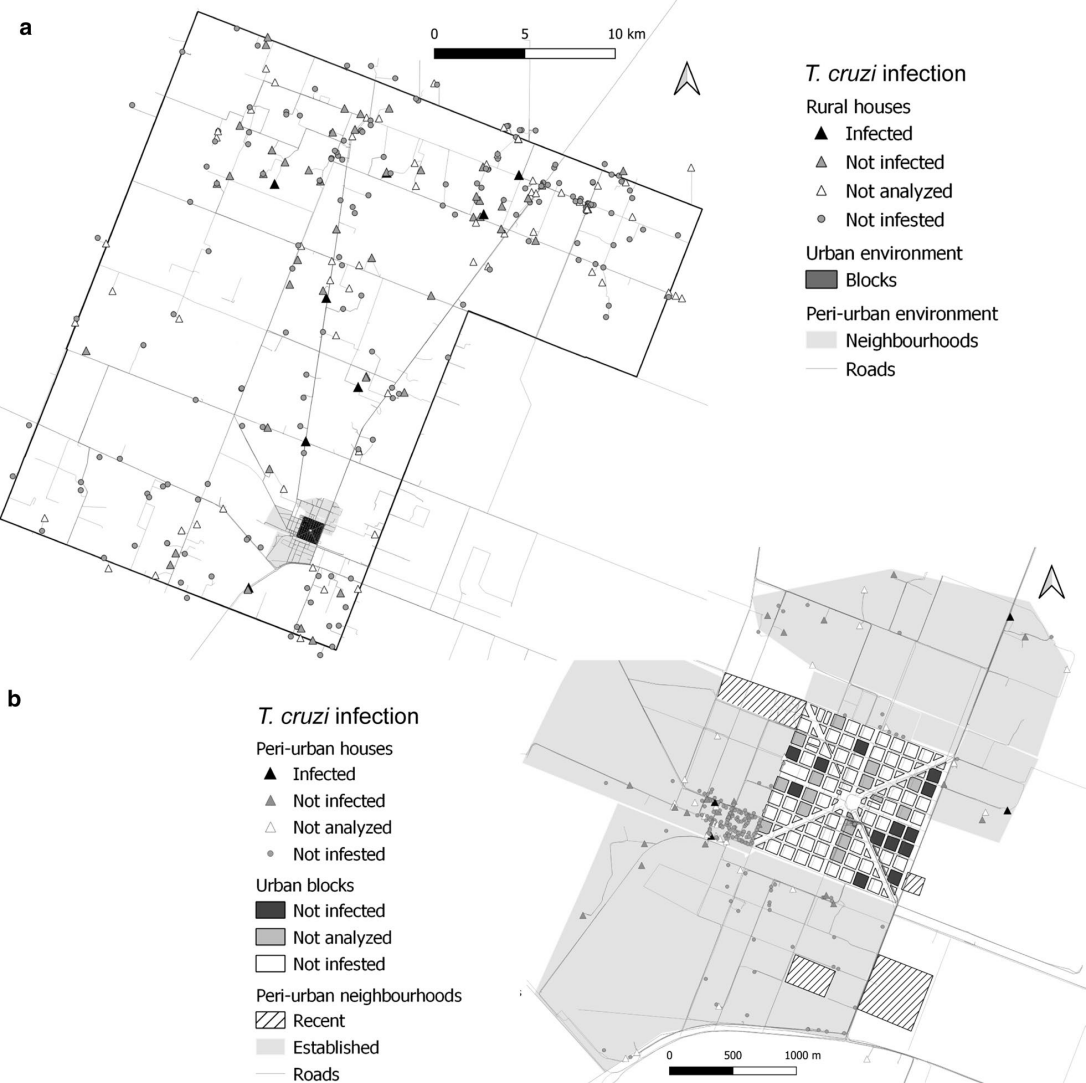
Spatial distribution of houses harboring *Trypanosoma cruzi*-infected and uninfected *Triatoma infestans*, Avia Terai, Chaco. ‘Not analyzed’ refers to infested houses where the triatomines collected in low-risk peridomestic ecotopes were not examined for infection. **a** Rural environment, **b** peri-urban and urban environments

Bug infection was found in 11.9% of 109 infested houses examined for infection. The percentage of infested houses with at least one infected bug in the peri-urban (16.1%) and rural (13.6%) environments was not significantly different (Fisher’s exact test:* df* = 1, *P* = 1.0) (Table 1); if all collected bugs had been examined, the adjusted prevalence of infection decreased to 8.2% (95% CI 6.7–24.4) and 7.9% (95% CI 3.2–12.8), respectively. Nearly half (53.8%) of the houses where infection was detected harbored only one infected triatomine. In houses with infested domiciles or high-risk peridomestic ecotopes, the mean number of *T. cruzi*-infected triatomines per house ranged from 0 (urban) to 0.5 (peri-urban) to 0.4 (rural). Only two rural houses harbored infected triatomines in more than one site within the house compound. No significant global or local spatial clustering of *T. cruzi* infection was detected in the peri-urban and rural environments (Fig. 1).

**Fig. 2:**
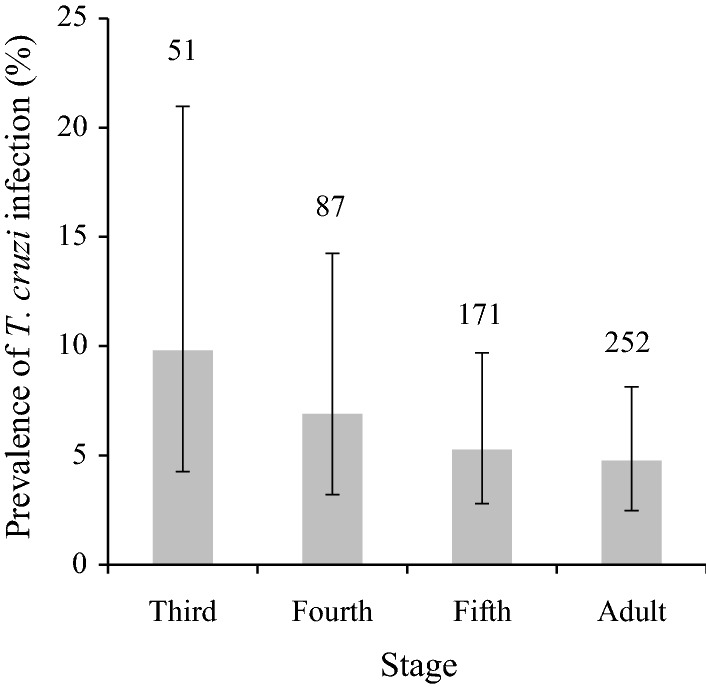
Stage-specific distribution of *T. cruzi* infection in *T. infestans*, Avia Terai, Chaco, 2015–2016. Numbers above the bars represent the number of triatomines analyzed for each stage

The number of triatomines examined for infection increased with higher bug stage across the rural-to-urban gradient, with adult bugs accounting for 44.9% of all examined triatomines (252/561 insects) (Fig. 2). There was no significant statistical difference in infection prevalence among bug stages for all types of environments combined (*χ*^2^ = 2.3,* df*  = 3, *P* = 0.5) (Fig. 2).

### Blood-feeding sources

Most triatomines examined for blood meal sources were reactive (85.4%) to at least one of the antisera tested (Additional file 1: Table S1). Reactivity (i.e. the percentage of ELISA-reactive bugs) differed significantly among environments (*χ*^2^ = 18.2,* df*  = 2, *P* < 0.001) and was significantly higher in peri-urban (93.3%) than in rural (85.6%) and urban environments (74.6%). Reactivity peaked in low-risk peridomestic ecotopes (95.2%) (Additional file 1: Table S1). Among reactive *T. infestans* with mixed blood meals (18.0%), 93.0% had fed on two different host species, and the remaining (7.0%) bugs were positive for three host species.

**Fig. 3:**
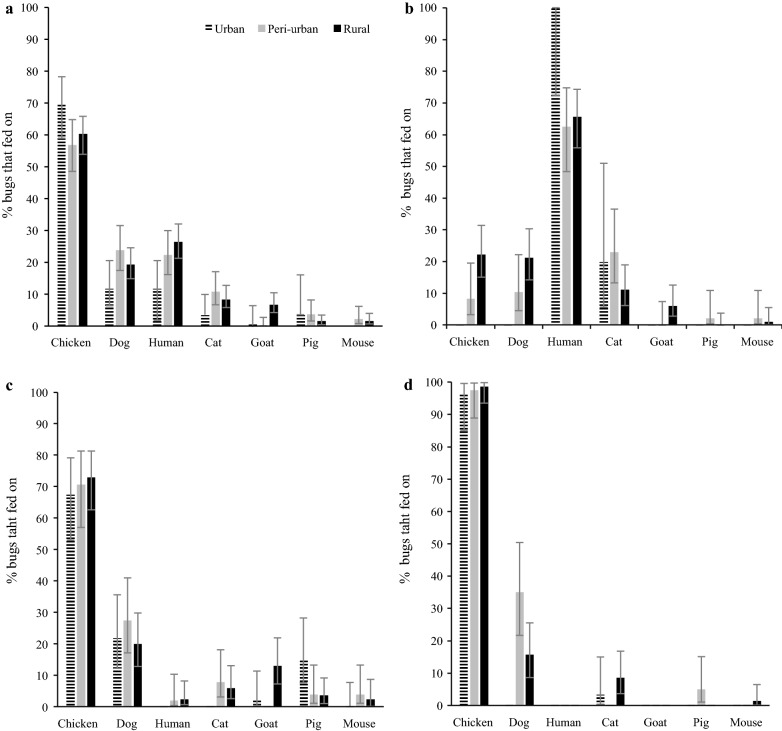
Distribution of bloodmeal sources in *T. infestans* by environment and ecotope, Avia Terai, Chaco, 2015–2016. Bugs were collected in: all ecotopes (**a**), domiciles (**b**), high-risk peridomestic ecotopes (**c**) and low-risk peridomestic ecotopes (**d**)

The most frequent blood meal source of *T. infestans* across environments was chickens, followed by humans and dogs (Fig. 3a). The rural environment was the only one that showed a full range of host types, having reactive samples for all the seven species tested (Fig. 3a). In domiciles across all environments, humans were the main blood meal source (urban: 100.0%, peri-urban: 62.5%, rural: 65.7%) and cats were the secondary source (20.0, 22.9, 11.1%, respectively) (Fig. 3b). Dogs and chickens were also secondary blood meal sources of domestic bugs in the peri-urban and rural environments: rural houses exhibited twice as many dog-fed domestic bugs (21.2%) than peri-urban ones (10.4%) (Fig. 3b). The percentage of chicken-fed domestic bugs was threefold higher in rural houses (22.2%) than in peri-urban ones (8.3%). The main blood meal source in peridomestic ecotopes was chickens, ranging from 67.4 to 98.6% of the reactive bugs across environments (Fig. 3c, d). Dogs were the secondary blood meal source in peridomestic ecotopes.

Cats, pigs, goats and mice contributed little as blood meal sources of peridomestic bugs. In the urban environment, pig (15.2%) and goat (2.2%) blood meals were detected in peridomestic ecotopes (Fig. 3). Pig blood meals were detected in four adult triatomines and three nymphs collected in a mud oven, a kennel and a woodpile from three urban houses having no pigs, although pigs were recorded in neighboring uninfested houses. The only goat-fed adult triatomine detected in the urban environment was collected in a house with no goats. However, a neighboring family had two infested houses, one in the urban environment and another in the rural one. In the rural house goat-fed bugs were detected.

### Human blood index

**Fig. 4:**
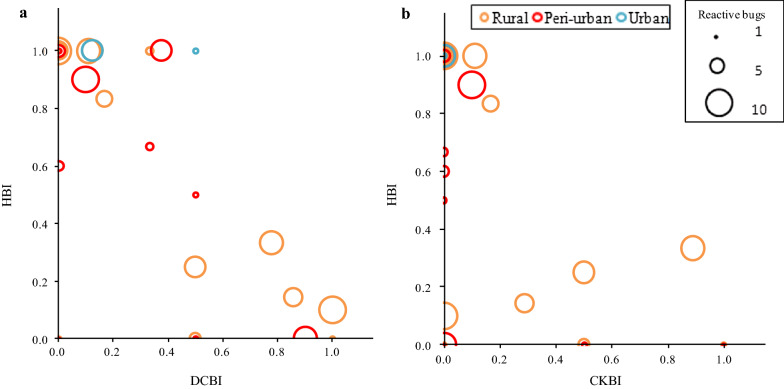
Proportion of domestic *T. infestans* that fed on humans (*HBI* [human blood index]) according to the proportion of domestic bugs that fed on dogs or cats (**a**) (*DCBI* [dog-cat blood index]) and chickens (**b**) (*CKBI* [chicken blood index]). Each data point corresponds to a different house. The size of the circles is proportional to the frequency of reactive bugs. Avia Terai, Chaco, 2015–2016

The HBI of domestic *T. infestans* peaked in the urban environment, where the few bugs examined contained human blood. No significant difference in HBI was found between rural (65.7%) and peri-urban domiciles (62.5%) (Fisher’s exact test:* df*  = 1, *P* = 0.7). The relative abundance of domestic bugs was not significantly associated with the HBI in the model clustered by house and adjusted for bug stage. The HBI significantly decreased with increasing proportions of dog- or cat- and chicken-fed bugs in a logistic regression (OR 0.004, 95% CI 0.00–0.13 and OR 0.03; 95% CI 0.00–0.86, respectively) (Additional file 2: Table S2; Fig. 4). The number of human residents exerted a significant positive effect on the HBI although with large uncertainty on its magnitude (OR 8.93, 95% CI 1.12–71.17 for domiciles with 4 or 5 humans). No significant effect of the household number of dogs or cats was detected (Hosmer–Lemeshow *χ*^2^ test = 8.88, *df*  = 8, *P* = 0.35). The type of environment was not included as a predictor in this model due to perfect separation of urban triatomines.

### Relationship between *T. cruzi* infection and blood meal sources

The blood meal source was identified in 87.5% of 32 *T. cruzi*-positive bugs. Mixed blood meals were twofold more frequent in infected bugs (31.3%; 95% CI 17.6–48.6%) than in uninfected ones (16.9%; 95% CI 13.7–20.6%). In peri-urban houses, the main blood meal sources of infected *T. infestans* were humans and dogs (infective blood meal indices: 29.0 and 12.1%, respectively), whereas in rural houses, cat-fed bugs displayed peak infective blood meal indices (27.3%) (Table 2). No infection was detected among bugs fed on goats, pigs or mice (i.e. “other”).

**Table 2:** Distribution of *T. cruzi* infection in *T. infestans* according to blood meal source, Avia Terai, Chaco, 2015–2016

Blood meal source	Infective blood meal index (no. reactive bugs)		
	Peri-urban environment	Rural environment	Total
Humans	29.0 (31)	9.0 (67)	15.3 (98)
Dogs	12.1 (33)	8.2 (49)	9.8 (82)
Cats	6.7 (15)	27.3 (22)	18.9 (37)
Chickens	1.3 (79)^a^	5.2 (153)^b^	3.9 (232)
Others	0.0 (6)	0.0 (24)	0.0 (30)

The infective blood meal index was estimated as the percentage of *T. cruzi*-infected bugs with an identified blood meal on host X relative to all bugs examined for *T. cruzi* infection that had an identified blood meal on host X

^a^All infected triatomines exhibited a mixed blood meal with dogs, cats and humans

^b^All infected triatomines exhibited a mixed blood meal with cats, with two exceptions

Firth-penalized multiple logistic regression showed that the relative odds ratio of *T. cruzi* infection was 16.6- and 13.2-fold greater for bugs in the peri-urban and rural environments, respectively, with respect to urban bugs, although confidence intervals were large. *Trypanosoma cruzi* infection in *T. infestans* collected in domiciles and high-risk peridomestic ecotopes was significantly associated with having a blood meal on humans, dogs or cats (Table 3). The relative odds ratio of having a *T. cruzi* infection was nearly three- to fourfold higher in bugs having a blood meal on humans (OR 3.15, 95% CI 1.27–8.17), dogs (OR 2.80, 95% CI 1.03–7.31) or cats (OR 4.02, 95% CI 1.46–10.39) than in bugs not fed on these hosts. In univariate analyses, the relative odds ratio of infection was significantly associated with the household number of dogs and cats and was ninefold higher in houses harboring > 8 dogs and cats than in houses with ≤ 3 cats and/or dogs (OR 9.49, 95% CI 3.49–31.66).

**Table 3:** Risk factors for *Trypanosoma cruzi* infection in *Triatoma infestans* collected in domiciles and high-risk peridomestic ecotopes, Avia Terai, Chaco

Predictor	Univariate analyses			Firth-penalized multiple logistic regression (*n* = 408)		
	Odds ratio	95% CI	*P*	Odds ratio	95% CI	*P*
Environment						
Urban	1.00	–	–	1.00	–	–
Peri-urban	20.81	2.67–2681.96	< 0.01*	16.60	2.07–2150.73	< 0.01*
Rural	16.10	2.17–2055.80	< 0.01*	13.23	1.73–1699.53	< 0.01*
Stage						
Third-fourth nymph	1.00	–	–	1.00	–	–
Fifth-instar nymph	0.67	0.26–1.65	0.38	0.63	0.24–1.65	0.35
Male	0.64	0.23–1.65	0.36	0.64	0.22–1.79	0.40
Female	0.45	0.15–1.26	0.13	0.39	0.12–1.12	0.08
Blood meal on						
Dog	1.85	0.77–4.10	0.16	2.80	1.03–7.31	0.04*
Cat	3.89	1.49–9.29	< 0.01*	4.02	1.46–10.39	< 0.01*
Human	2.69	1.29–5.54	< 0.01*	3.15	1.27–8.17	0.01*
Chicken	0.64	0.28–1.35	0.25	1.13	0.43–2.88	0.80
Site bug abundance (triatomines captured per one-person h)						
≤ 8	1.00	–	–	–	–	–
9–20	1.34	0.44–4.71	0.62	–	–	–
21–52	0.83	0.27–2.99	0.77	–	–	–
> 52	0.49	0.15–1.76	0.25	–	–	–
Number of dogs and cats per house^a^						
≤ 3	1.00	–	–	–	–	–
4–5	0.76	0.13–3.50	0.73	–	–	–
6–8	2.21	0.71–7.86	0.17	–	–	–
> 8	9.49	3.49–31.66	< 0.01*	–	–	–
Number of human residents per house^b^						
≤ 2	1.00	–	–	–	–	–
3	0.57	0.14–1.71	0.34	–	–	–
4–7	0.17	0.03–0.55	< 0.01*	–	–	–
> 7	0.60	0.23–1.44	0.26	–	–	–

*Significant at* P *≤ 0.05

^a^The number of dogs and cats was unknown for 5 examined bugs

^b^The number of residents was unknown for 98 examined bugs

## Discussion

Based on the prevalence of *T. cruzi* infection in triatomines and on human–vector contact rates measured by HBI (the risk components examined in this study), infested peri-urban and rural houses would be under a similar risk of transmission if other risk components (e.g. prevalence of human, cat and dog infection) were equal among environments. These patterns partially contradict the expected decrease in the prevalence of bug infection across the rural-to-urban gradient given the decreasing trends in house infestation and bug abundance. Our study reveals (peri)domestic *T. cruzi* transmission was likely occurring in peri-urban and rural houses from Avia Terai municipality 2–4 years after community-wide residual spraying with pyrethroid insecticides. Unexpectedly, infested urban houses had no infected *T. infestans* despite frequent occurrence of human infection and human–vector contact in the few domestic bugs collected (see below).

*Trypanosoma cruzi* infection was detected in 2% of all triatomines and in 8% of the infested houses. Lack of spatial aggregation of *T. cruzi-*infected bugs suggests independent transmission foci. These results evince a low prevalence but spatially spread distribution of *T. cruzi* infection in *T. infestans* in the context of rather recent insecticide applications by vector control personnel. For example, widespread bug infection in peri-urban scenarios were also reported for San Juan Province, western Argentina [42], in Chaco province in the 1940s [43] and in the outskirts of Cochabamba [17]. In contrast, *T. cruzi* infection in *T. infestans* in peri-urban Arequipa was spatially aggregated and restricted to certain locations [10]. Spatially spread house infestation and *T. cruzi* infection encompassing peri-urban areas, as in Avia Terai, pose additional challenges to vector elimination efforts originally conceived for rural areas. In addition to the much higher number of target houses and insensitivity of triatomine detection methods, the occurrence of closed or nonparticipating households may hinder access to premises in peri-urban and urban areas.

In our study, peri-urban and rural environments had a similar prevalence of *T. cruzi* infection and similar percentages of infested domiciles (21–24%) and high-risk peridomestic ecotopes (15–18%) harboring infected bugs. These patterns remained despite rural houses having higher house infestation and higher bug abundance. This can be related to the high proportional contribution of low-risk peridomestic ecotopes to rural infestation and bug abundance [25] (Table 1), but in which no infected bug was detected.

The main blood meal source detected in domestic bugs was humans, whereas chickens and dogs were the main blood meal sources in the peridomestic ecotopes, as in other settings [27, 28, 44–47]. Domestic *T. infestans* collected in the Paraguayan Chaco nearly exclusively fed on humans [48], in a context of strong vector control activities. In Avia Terai, chickens were relevant blood meal sources in all ecotopes across the rural-to-urban gradient, with the exception of urban domiciles where we found bugs only fed on humans and cats. Chicken-fed bugs in domiciles are intimately linked to the widespread rural practice in the Chaco region of letting hens nest indoors for protection, especially in the spring and summer [19, 49], which corresponds to the timing of the current survey.

Cats apparently played a different epidemiological role across the gradient, serving as a highly infectious and frequent blood meal source in rural houses and urban domiciles, respectively, while contributing little in peri-urban settings. These results are probably related to variations in domestic host abundance [25] and domestic animal management practices across the gradient.

The host-feeding patterns of *T. infestans* and other major triatomine vectors usually correlate closely with the main local resident host(s) in the collection ecotope [18]. The main blood meal sources identified in this study matched closely the* a priori* classification of ecotopes based on the expected occurrence of infected hosts. A high frequency of chicken-fed bugs was recorded in high-risk peridomestic ecotopes, and 31 (22%) insects from low-risk peridomestic ecotopes in rural and peri-urban houses had mixed blood meals on chickens and dogs or cats. These results point to the high mobility of triatomines and unconfined hosts. We also registered a wide diversity of blood meal sources in urban and peri-urban environments, including pigs and goats. Long-standing rural practices explain the presence of domestic ungulates in peri-urban and urban settings [25]. Additionally, active bug dispersal and passive bug transport from the rural environment may explain the few blood meals on host species not recorded at the bug collection house. Although most urban infestations consisted of triatomine colonies (88%) rather than invading adult triatomines [25], short distances between neighboring houses favor triatomine dispersal, as in Arequipa [50]. Frequent shifts from the refractory chickens or other uninfected hosts to dogs or cats, which frequently are infected and highly infectious, increase the risk of transmission [27, 51].

In our study, the urban environment had significantly fewer reactive triatomines than did rural and peri-urban ones. Bugs may have less access to blood-feeding in the urban environment due to a lower abundance of hosts, fewer peridomestic structures and/or better housing quality and animal management practices compared to rural and peri-urban environments [25, 52]. These specific factors observed in urban houses may also explain the unexpected lack of bug infection. Further investigations on *T. infestans* feeding frequency across the rural-to-urban gradient may shed light on this issue.

Domestic triatomines in urban settings displayed the highest HBI (100%), the lowest prevalence of mixed blood meals (6%) and the absence of dog-fed bugs. These results may be related to urban householders restraining domestic animals, particularly dogs, from entering domiciles more frequently than peri-urban and rural dwellers. None of the two urban households with infested domiciles reported allowing dogs inside, whereas this occurred in 14% of infested peri-urban domiciles and in 27% of rural ones. Specifically designed studies are needed to confirm these findings since data on animal resting sites were missing for many houses.

Peri-urban and rural houses had similar frequencies of domestic bugs with mixed blood meals (19 and 22%) and HBI (62 and 65%), respectively. As expected, the proportions of dog- or cat-fed bugs (dog-cat blood index) and of chicken-fed bugs (chicken blood index) significantly decreased the HBI of domestic *T. infestans* [27, 28, 45]. These results further support the hypothesis that the occurrence of domestic animals indoors strongly affects human–vector contact rates and triatomine infection.

In contrast to our initial hypothesis, the HBI was not associated with the number of dogs or cats in a household in the multivariate analyses, which considered the absolute number of chickens, dogs and cats in each house instead of those actually resting or nesting indoors. Another limitation of our study was the lack of data on human overcrowding and other direct measures of host density more directly related to local host availability. These limitations hamper the estimation of the number of available hosts indoors and its relationship with transmission components. One strength of the current study is the molecular diagnosis of *T. cruzi* infection in a large sample of triatomines selected carefully across an environmental gradient. Molecular methods enhance the detection of light infections, a fraction of which are usually missed by optical microscopy. The false negative rate reached 10–13% in xenodiagnosis bugs examined by optical microscopy [53]. An additional advantage of using molecular diagnostic methods was the immediate freezing of collected triatomines upon completion of daily fieldwork. This at least partially explains the high reactivity of blood meal identification tests regardless of where the insects had been collected.

Triatomines that fed on humans in peri-urban houses and on cats in rural ones displayed high infective blood meal indices. Humans, dogs and cats contributed significantly to *T. infestans* infection in Avia Terai. Nearly 22–24% of urban households in Avia Terai reportedly harbored at least one *T. cruzi*-infected resident in 2012 [54]. A human serosurvey involving > 1000 people conducted in 2017 across the gradient showed that nearly 15% were seropositive for *T. cruzi* (Cardinal et al., unpublished). Hence, humans are also a potential source of *T. cruzi*. Although no data on the seroprevalence of *T. cruzi* infection in dogs and cats are currently available, the infective blood meal indices in our study suggest that infected dogs and cats were likely present.

## Conclusions

The results of our study reveal that active transmission was likely occurring in peri-urban and rural settings of Avia Terai. Despite no infected bug being found in urban houses, the high human–vector contact detected, the occurrence of low-density domestic colonies, widespread human *T. cruzi* infection and high density of households and hosts set a favorable scenario for vector-borne transmission if domestic infestations are allowed to thrive. In endemic countries, densely populated urban and peri-urban areas represent an unexpected challenge for vector control programs, mainly conceived for rural environments. Tailored vector control activities adapted to peri-urban and urban environments, strong community mobilization and sustained vector surveillance are required to effectively suppress the risk of domestic vector-borne transmission of *T. cruzi* and eliminate *T. infestans*.

## Supplementary Information

**Additional file 1: Table S1.** Distribution of bloodmeal reactivity in *T. infestans* according to type of ecotope and environment.

**Additional file 2: Table S2.** Relationships between human blood index and selected factors in domestic *T. infestans*, Avia Terai, 2015–2016.

**Additional file 3: Table S3.** Dataset including infection status, bloodmeal source identification, environmental and demographic variables, Avia Terai, Chaco, 2015–2016.

## Data Availability

The dataset supporting the conclusions of this article is included within the article and in the Additional files. The dataset generated during and/or analyzed during the present study are available in Additional file 3: Table S3.

## Abbreviations

HR: High risk
LR: Low risk
