# Supplementary material for: Trypanosoma cruzi infection in Triatoma infestans and high levels of human–vector contact across a rural-to-urban gradient in the Argentine Chaco
Source: Parasit Vectors. 2021 Jan 9;14:35. doi: 10.1186/s13071-020-04534-z (PMC7796388; doi:10.1186/s13071-020-04534-z)
Supplement: Supplementary file 1 — Additional file 1: Table S1. Distribution of bloodmeal reactivity in T. infestans according to type of ecotope and environment. [file 13071_2020_4534_MOESM1_ESM.docx]

**Table S1.** Distribution of bloodmeal identification reactivity in *T. infestans* according to type of ecotope and environment.

|  | % of reactive bugs (no. examined) | | | | % of bugs with mixed blood meals |
| --- | --- | --- | --- | --- | --- |
| Ecotope | Urban | Peri-urban | Rural | Total |  |
| Domiciles | 83.3 (12) | 90.6 (52) | 85.3 (116) | 87.2 (180) | 19.8 |
| Peridomestic HR | 69.1 (67) | 92.7 (55) | 81.1 (106) | 80.3 (228) | 14.2 |
| Peridomestic LR | 81.8 (35) | 95.2 (42) | 91.3 (76) | 90.6 (153) | 20.9 |
| Total | 74.6 (114) | 93.3 (149) | 85.6 (298) | 85.4 (561) | 18.0 |

Peridomestic HR: peridomestic ecotopes with high-risk of infection.

Peridomestic LR: peridomestic ecotopes with low-risk of infection.
