# Supplementary material for: Trypanosoma cruzi infection in Triatoma infestans and high levels of human–vector contact across a rural-to-urban gradient in the Argentine Chaco
Source: Parasit Vectors. 2021 Jan 9;14:35. doi: 10.1186/s13071-020-04534-z (PMC7796388; doi:10.1186/s13071-020-04534-z)
Supplement: Supplementary file 2 — Additional file 2: Table S2. Relationships between human blood index and selected factors in domestic T. infestans, Avia Terai, 2015–2016. [file 13071_2020_4534_MOESM2_ESM.docx]

Table S2. Relationships between human blood index and selected factors in domestic *T. infestans*, Avia Terai, 2015-2016.

| Predictor | OR | 95% CI | *P* |
| --- | --- | --- | --- |
| Stage |  |  |  |
| Third-fourth instar nymph | 1.00 | – | - |
| Fifth-instar nymph | 2.59 | 0.42 – 15.89 | 0.30 |
| Male | 0.16 | 0.02 – 1.55 | 0.11 |
| Female | 0.75 | 0.10 – 5.40 | 0.78 |
| Domestic bug abundance (triatomines captured per one-person hour) | | | |
| ≤1 | 1.00 | – | - |
| 2-3 | 2.96 | 0.15 – 58.41 | 0.48 |
| 4-10 | 6.14 | 0.47 – 80.23 | 0.17 |
| >10 | 2.94 | 0.22 – 39.20 | 0.42 |
| Number of humans per house | | | |
| 1 | 1.00 | – | - |
| 2-3 | 6.93 | 0.76 – 63.53 | 0.09 |
| 4-5 | 8.93 | 1.12 –71.17 | **0.04** |
| >5 | 8.32 | 0.70 – 63.53 | 0.09 |
| Number of dogs and cats per house | | | |
| ≤4 | 1.00 | – | - |
| >4 | 0.61 | 0.11 – 3.37 | 0.57 |
| DCBI | 0.00 | 0.00 – 0.13 | **<0.01** |
| CKBI | 0.03 | 0.00 – 0.86 | **0.04** |

DCBI: dog or cat blood index, CKBI: chicken blood index.
